# Inspirational Leaders in Surgery: Dr. Haile Debas

**DOI:** 10.1007/s00268-022-06680-0

**Published:** 2022-07-29

**Authors:** Doruk Ozgediz

**Affiliations:** Department of Surgery, Division of Pediatric Surgery, UCSF Center for Health Equity in Surgery and Anesthesia, San Francisco, USA


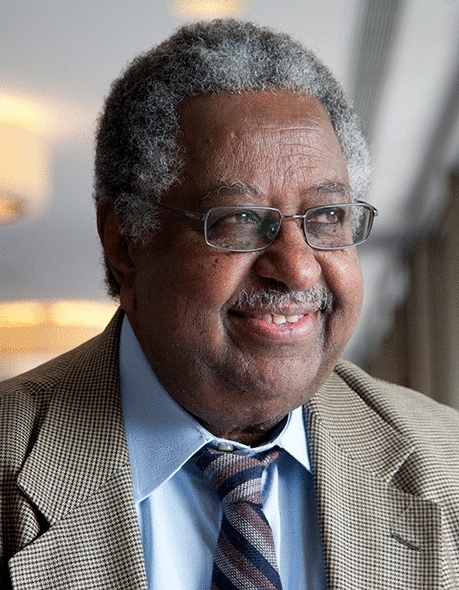
“Dr. Debas, we know it’s midnight and you just arrived here, but the hospital needs you now,” said the Royal Canadian Mountain Police officer over the phone. Dr. Debas and his wife Kim had just arrived in Whitehorse, the Yukon Territories, where he was to replace the vacationing sole general surgeon in this remote part of Canada in 1970. The patient in question had significant post-partum hemorrhage 3 days prior, and the uterus had been packed—she was now septic. He knew there was no time to waste, brushed up on essential anatomy for emergency hysterectomy, and immediately treated a ruptured uterus in this rural hospital, saving this patient’s life. “It is one of the great things about surgery, that you can really make a difference in people’s lives,” he recalls.

It was an unlikely place for this giant in academic surgery to be, but one in a twist of unexpected and inspiring turns in a life and storied career marked by challenges turned into opportunities.

Haile T. Debas was born in 1937 in Asmara, Eritrea, where his father was a government official. He attended elementary school there before moving to Tigray, Ethiopia to be with his brother. He then entered the Wingate secondary school in Addis Ababa, an English school where he “got the best grounding in my education.” From there, he entered the University College of Addis Ababa where he graduated with a Bachelor of Science degree. His first love was physics, but his relatives and friends convinced him that he would “end up as a high school teacher,” and encouraged him to consider medicine.

At that time, there was no medical school in Ethiopia, and Eritrea and Ethiopia were one country under Haile Selassie. While most students went to the American University of Beirut, Dr. Debas was accepted to McGill University in 1963 where “we had fabulous teachers who inspired us in all fields.” In particular there was a new Chair of Surgery, Lloyd MacLean who “showed me that you could be an excellent clinical surgeon, a wonderful educator, and a very significant investigator all at once. That told me I should do surgery. Also I thought, since I was going back to Ethiopia, I might be more useful as a surgeon than if I trained in other areas.” (1) This was followed by surgical residency in Vancouver, which was “a tremendous clinical experience” that also included a gastrointestinal physiology research scholarship in Glasgow, Scotland.

He had been promised a faculty position at Haile Selassie University in Addis Ababa upon completing general surgical training. After finishing residency, Dr. Debas married Kimfah Ignacia Assing (Kim) of Trinidad, and together they moved to Whitehorse, Yukon, where Dr. Debas had accepted a locum tenens position as a general surgeon to earn some extra money before returning to Ethiopia. While there, he received a telegram from the Ethiopian Government explaining that the position he was promised was no longer available. He was shocked and disappointed, and this presented one of the greatest challenges of his career. “I really didn’t know what to do,” he recalled.

He immediately tried to get a Canadian visa—was initially denied—but due to Kim’s Canadian citizenship—was able to get one. Then, there was finding a job. There was no immediate job in Vancouver, but there was a surgical need in rural, Northern British Columbia, which is how he ended up at Burns Lake (-40 degrees Celsius in the winter). “In those days we had training in obstetrics, neurosurgery, orthopedics and those came in very handy as a rural surgeon.” He recalled an additional case when the town mayor came, in the middle of a snowstorm, with severe cholangitis who Dr. Debas treated successfully. Seeing this, the hospital then asked him to stay to develop a surgical practice, and they gave him a very generous package to buy surgical equipment. “I went to Edmonton with Kim, to a medical supply store, and like a kid in a candy shop, I bought everything I wanted.”

While in Burns Lake, he recalls, “I contemplated my career and realized I would not be competitive for a career in academic surgery in North America.” He wrote to the leading GI physiology researcher in the world—Dr. Morton Grossman at UCLA. “This took a lot of chutzpah- and I didn’t expect any response, so I was shocked when he wrote back and said, ‘Yes you can come but I have no money for you’.” Dr. Debas obtained a research fellowship and went to UCLA in 1972 for two years to work with Dr. Grossman, and says “that was probably the most important thing that I did professionally, as it opened a lot of doors for me.”(2).

Indeed, many doors opened for Dr. Debas, including faculty positions at the University of British Columbia and UCLA, and leadership of the Division of Gastrointestinal Surgery at the University of Washington, where he continued advancing the field of gastrointestinal physiology. He would then go on to lead the Department of Surgery at the University of California, San Francisco (UCSF), becoming the first African chair of an American Department of Surgery, and catalyzing the clinical and scientific growth of the department. His subsequent leadership positions at UCSF would include Dean of the Medical School, where he pioneered significant advances in medical education, and Chancellor.

He highlighted another big career challenge in leadership, which was the UCSF-Stanford merger that dissolved, a situation Dr. Debas inherited as Dean and Chancellor of UCSF. “I had to make sure that UCSF recovered from the demerger. After the demerger both places lacked senior leadership, and both were in debt.” He recalls the recruitment of Mark Laret to lead the UCSF hospital as key success in the recovery.

In 2003, Dr. Debas stepped down as Dean, planning to return to Eritrea to start a medical school. However, politics interfered again as democratic elections were canceled. Dr. Debas was among 13 prominent expatriate Eritreans advocating directly to the President for democracy, but this plea was unsuccessful, and he could not return to his home country to advance medicine in the way he dreamed. “I decided to re-invent myself,” he recalls, “and we started a Global Health Sciences Institute at UCSF.” While this was easy to do at UCSF, initiating a ten campus University of California entity, which he also undertook, was very difficult to maintain due to uncertain resources. Eventually, it landed on solid ground with university funding, but the journey was difficult.

Reflecting on the legacies of his career, Dr. Debas shares “the students, residents and fellows, especially the fellows–they become part of your extended family. It really is a great privilege to train residents and fellows.” (3) He also sees that “I got interested in global health early, at a time when surgery was not considered a global health endeavor, I worked hard to get it on the agenda and take it to the next level.” (4, 5).

He sees the erosion of the patient doctor relationship as the most significant issue the surgical community needs to address. “The patient doctor interaction has diminished greatly, but in surgery maybe more than any other area, the patient-doctor relationship is most critical because the patients put their lives in your hands. There is less attention to listening to and examining the patient, and somehow, we have to restore that.” He pays tribute to the American College of Surgeons for their focus on excellence in surgical education, “such an important thing for the house of surgery” citing the impact of Drs. Ajit Sachdeva and L.D. Britt and the development of the Academy of Master Surgical Educators.(6).

He also shares, “It’s very important that surgery does not lose its focus on research. Surgeons have a lot to contribute to all relevant fields. For our residents, it fosters critical thinking. Surgery also has to be seen as a great advocate for patients who should see us as their champions.”

For trainees and young surgeons, he shares, “Don’t be afraid to aim for the best, because you have it in you and you may not know it. From my own life experience, when there are challenges that’s when the opportunities exist. See how you can reinvent yourself. There is always another path. And remember that anything you achieve, you don’t do it by yourself. Humility and giving credit to others is a very important aspect of your career.”

He also cautions, “We surgeons are so involved in our profession we may not pay attention to creating balance in our lives, I hold myself as an example of that. Thank God the younger generation has paid more attention to promoting wellness and avoiding burnout. It’s a very important challenge for us.” Dr. Debas shares that he and his wife adopted two grand-nieces who are now in North America, and his joy in grandparenting. He concludes about his wife Kim, “It’s a great privilege to have a spouse who understands a surgeon’s life, as we put a great demand on our spouse’s and our families’ lives – this was both when I was a busy clinical surgeon, and I was an administrator. I am blessed to have a partner who made all this possible for me.”
